# Is intrauterine growth appropriate to monitor postnatal growth of preterm neonates?

**DOI:** 10.1186/1471-2431-14-14

**Published:** 2014-01-17

**Authors:** Luis Pereira-da-Silva, Daniel Virella

**Affiliations:** 1 Neonatal Intensive Care Unit, Hospital Dona Estefânia, Centro Hospitalar de Lisboa Central, Rua Jacinta Marto, 1169-045 Lisbon, Portugal

**Keywords:** Intrauterine charts, Crown-heel length, Measurements, Postnatal growth, Preterm

## Abstract

When using the useful 2013 Fenton Chart, data should be interpreted with caution taking into account two aspects: the physiologic loss of body water after birth for the weight curves, and the questionable accuracy of the birth length curves considering the heterogeneity and reliability of the methods used in the original measurements.

## Background

The revised 2013 Fenton Preterm Growth Chart harmonized a very large meta-analysis of size at birth for preterm with the new World Health Organization Growth Standard [1]. This chart is intended to provide a single useful tool for different purposes in preterm infants: assessing intrauterine growth at birth, monitoring postnatal growth up to term and monitoring growth after term. The 2013 Fenton Chart represents reference curves closer to normative standard, considering that reference charts describes how growth actually is, while standard indicates how growth should be [2]. We consider that when using 2013 Fenton Chart, the plotted data should be interpreted with caution regarding two important aspects: the physiologic loss of body water after birth and the reliability of the crown-heel length curves.

### Postnatal use of intrauterine growth data

The American Academy of Pediatrics [3] and the Canadian Pediatric Society [4] recommend that preterm infant growth should approximate intrauterine growth, with the argument that the fetus is not affected by extrauterine factors with negative impact on the nutrition status and growth, such as suboptimal nutrition support, major neonatal complications and medical interventions that increase energy expenditure and nutrient losses [5]. However, the application of intrauterine growth rates to preterm infants in an extrauterine environment may be inadequate during the first postnatal weeks [6] and even during the whole neonatal period. Loss of body water is an integral part of the physiology of postnatal adaptation and largely accounts for normal weight loss after birth [7]. This does not occur in the fetus. Even providing the better current nutritional support to a “healthy” premature neonate, after normal body water loss has occurred, it is expected that a significant lag will be established between the rising extrauterine growth curve and the growth curve of a fetus of similar gestational age; in general, the postnatal weight curve will parallel and not exceed the intrauterine curve, maintaining the mentioned lag [8]. Proposing the intrauterine growth as a goal for preterm infants may not be realistic and may be physiologically biased. This assumes that following the initial weight loss, the weight gain should reach the intrauterine growth curve reflecting a recovery of fat and muscle mass lost after birth, despite body weight loss in this period being predominantly due to physiologic extracellular water loss [9]. Attempting to mimic intrauterine growth in early postnatal life may be achieved with excessive increase in fat mass, predisposing to obesity and late metabolic syndrome [10]. Therefore, intrauterine growth data derived from cross-sectional measurements of birth weight overestimate postnatal growth, are not representative of the physiology of the neonates of the same corrected gestational age, and may not be ideal for monitoring growth and guiding nutritional support in preterm infants. At present, the available postnatal longitudinal growth charts for preterm infants are essentially a descriptive reference, accounting for the physiologic postnatal water loss, but influenced by nutrition practices contemporary to the construction of the charts, possibly outdated, as the 1999 Ehrenkranz chart [8]. The new standards that are being developed by the International Fetal and Newborn Growth Consortium for the 21st Century study may provide better reference curves closer to standard, designed from longitudinal data of a selected population of preterm neonates with the lowest risk for factors known to affect prenatal or postnatal growth [2].

### Reliability of the length curves

The second concern is related with the 2013 Fenton crown-heel length curves based on two [11,12] of the six surveys on size at preterm birth included in the meta-analysis. The small dimension of the samples providing birth length data in comparison with those providing birth weight data may be due to the methodological difficulty in obtaining accurate neonatal length measurements in large multicenter surveys. Measured neonatal length may be influenced by reluctance of the observer to provoke discomfort when extending the lower limbs against the normal flexor posture, especially in term infants [13]. Moreover, it seems that tape was used for measuring the length in the survey by Olsen et al. [11], the greatest sample of preterm neonates with measured birth length. Measurements are less reliable when using inappropriate instruments, such as measuring tape [14]. Charts based on less reliable data have limited accuracy [15], and their inclusion in meta-analyses contributes to unaccountable heterogeneity, being methodologically arguable [16].

## Conclusions

The use of the more recent descriptive curves seem more realistic and appropriate than intrauterine growth data for monitoring postnatal growth, while reference curves closer to standard are not released. In fact, there is no evidence for either the benefit or the safety of using intrauterine growth pattern to guide the nutritional support in preterm infants during the first postnatal weeks. Individual data should be interpreted with caution when plotting crown-heel length on charts based on original measurements that have been obtained without the recommended technique.

## Response

### Intrauterine growth references are appropriate to monitor postnatal growth of preterm neonates

Tanis R Fenton and Jae H Kim

(http://mailto:tfenton@ucalgary.ca)

### Abstract

No growth chart has resolved what to do with the physiologic postnatal weight loss when extracellular water is decreased. Nevertheless, the Fenton Growth Chart provides the most comprehensive comparison to the current growth standard for the preterm infant particularly with growth after return to birthweight. Existing postnatal growth references have limitations since they are based on less than ideal samples, and do not provide guidance about the importance of any deviations from the mean of the reference samples. Errors in length measurement have been found to be evenly distributed as under and overestimations, and therefore are unlikely to introduce bias.

**Keywords:** Intrauterine charts, Crown-heel length, Measurements, Postnatal growth, Preterm

We would like to thank Dr. Pereira-da-Silva and Dr. Virella for their insightful comments and thoughtful endorsement of our revised growth chart [1]. We agree with them about their concerns, with qualifications.

### Postnatal use of intrauterine growth data

Drs. Pereira-da-Silva and Virella expressed valid concerns regarding the differences between the growth pattern of preterm infant and that of intrauterine growth, particularly during the first postnatal weeks, due at least in part to the postnatal physiologic weight loss. We agree that this loss cannot be readily accounted for in intrauterine growth data. It has been estimated that this initial loss is primarily due to water loss from a reduction of extracellular volume (ECV) from an expanded ECV in the fetus [7]. While it could be argued that before birthweight is regained, some of this loss may include some lean body mass loss due to missing the rapid growth of the fetus, indirect calorimetry measurements suggest that even in ventilated infants energy expenditures can be met by parenteral and enteral support [17] and nitrogen retention can be achieved [18-20] even in the first days of life. Whether it is more appropriate to reassign a new z-score trajectory target once they decrease their ECV for their post-natal environment, or whether they should return to their birth z-score trajectory, remains a theoretical question. Therefore using a weight gain trajectory beginning post ECV loss, with guidance provided by the distribution of the fetus, is the most appropriate goal for preterm infants to follow at present until a more representative and validated growth reference can supersede this.

The primary alternative to growth monitoring by our type of Fetal-infant chart is to use longitudinal postnatal growth patterns of other preterm infants. Using other preterm infants as a growth reference has several critical limitations, including: a) the changing growth pattern as preterm infant nutrition and medical care improves [21], b) the representation of only the mean growth pattern, c) inclusion of heterogeneous populations of normal and abnormal infants (with growth restriction and/or various morbidities) in most growth references, and d) the lack of reference to intrauterine fetal [4,22] and term infant [4,22] growth which are the growth standards. Existing postnatal growth references do not provide guidance about the importance of any deviations from the mean of the reference sample. For example, if a preterm infant is growing faster than the reference preterm infant mean by 100, 500, or 1000 grams at 60 days of age, is this superior or excessive growth? This question is best addressed by assessing the growth relative to fetal and term infant growth, which can be done using the distributions on growth charts such as ours [1]. Our PreM Growth study adds support to this assessment since among infants born at 24 weeks, infants were as much as 800 grams at 60 days and 1160 grams at 100 days greater than the mean of their cohort, while remaining within the outer curves, the 3rd and 97th centiles. Further, a comparison of our PreM Growth study with the National Institute of Child Health and Human Development Neonatal Research Network large survey of infants conducted a decade prior [8] revealed a statistically significant improvement in the time to regain birthweight, with a resulting change of the growth pattern [21].

### Reliability of the length curves

In contrast to the concern raised about heterogeneity of the included length surveys behind our growth charts, our Figure three illustrating the length measurement distributions from the included American and Italian studies [1] does not suggest important heterogeneity, and the 3rd and 50th centiles showed remarkable agreement.

We agree there are likely to be some random and systematic errors in population-based newborn length surveys. Random errors are likely introduced due to imperfect measurement techniques (such as using a tape measure and measurements made without a length-board), as well as some systematic errors, due to normal flexor posture of term and near-term infants. A comparison of 602 term newborn measurements comparing the midwives measurements with those of two trained people using a length-board and standardized technique found the errors to be evenly distributed as both under and overestimations, which suggests that the errors in length measurement are random and not systematic [23]. Therefore since random errors vary in both directions of over- and underestimation, the random errors in large population-based surveys are likely to provide an unbiased result.

The high tone flexor posture with difficulty straightening infants’ legs are likely minor at early gestational ages when tone is low, increasing with gestational age to peak around term. These errors would be a concern among both infants in the NICU setting as well in the reference surveys, as a part of the reason why a length-board and two trained measurers are recommended [23]. The systematic errors due to tone near term age are likely similar between the reference curves and neonatal intensive care measurements. Of most importance is making accurate length measurements of infants being monitored in the NICU, so that the growth trajectory of the individual infant can be accurately assessed. Wood et al. found that using a length board, which enables reliable infant length measurements, is easy to use, accurate, inexpensive, and easy to teach others to use reliably in one hour [23].

Until we have a large survey of length measurements among preterm newborns, at all gestational ages of preterm infant survival, using ideal measurement technique, we will only have imperfect estimates. The widely anticipated studies, the International Fetal and Newborn Growth Consortium (INTERGROWTH-21st) study (designed with detailed inclusion and exclusion criteria with standardized nutrition and neonatal care strategies) and the National Standard for Normal Fetal Growth study (based on healthy low-risk pregnant women) may overcome the limitations of previous preterm postnatal growth studies [24].

### Further comments/ In conclusion

Intrauterine birth size data, although imperfect, is currently the normative standard for the assignment of size for gestational age [4,22]. Until this next generation of growth charts are produced, we believe that our 2013 growth charts for preterm infants, based on the recommended growth standard for preterm infants (the fetus [4,22] and the term infant [4,22], prepared using strict inclusion criteria for the large sample size meta-analysis of almost 4 million babies, combined with the World Health Organization Growth Standard, with smoothing informed by an analysis of preterm infant growth [21], is the superior chart currently available for monitoring growth of preterm infants. We would argue that even after the INTERGROWTH-21st growth charts [24] are available, there may still be a use for both postnatal longitudinal and intrauterine-based growth charts to assess the growth of preterm infants relative to their the normative standard.

## Competing interests

The authors declare that they have no competing interests.

## Authors’ contributions

LPdS contributed in the conception and drafting of the manuscript. DV contributed in the critical review of the manuscript. Both authors have given final approval of the version to be published. TRF drafted the response with assistance from JHK. Both authors have given final approval of the version to be published.

## Authors’ information

LPdS, MD, PhD: senior pediatrician and neonatologist; and professor of pediatrics. DV, MD, MSc Epi: senior pediatrician and neonatologist; and epidemiologist. Both authors’ affiliation: tertiary NICU, Hospital Dona Estefânia, Centro Hospitalar de Lisboa Central, Lisbon, Portugal.

TRF, RD, PhD: dietitian and epidemiologist, Alberta Children’s Hospital Research Institute, Institute of Population and Public Health, Department of Community Health Sciences, University of Calgary, Calgary, AB, Canada.

JHK, MD, PhD: neonatologist and pediatric gastroenterologist, Divisions of Neonatology & Pediatric Gastroenterology, Hepatology and Nutrition, UC San Diego Medical Center & Rady Children’s Hospital of San Diego, San Diego, CA, USA.

## Pre-publication history

The pre-publication history for this paper can be accessed here:

http://www.biomedcentral.com/1471-2431/14/14/prepub
